# A Fully Implantable Wireless ECoG 128-Channel Recording Device for Human Brain–Machine Interfaces: W-HERBS

**DOI:** 10.3389/fnins.2018.00511

**Published:** 2018-07-30

**Authors:** Kojiro Matsushita, Masayuki Hirata, Takafumi Suzuki, Hiroshi Ando, Takeshi Yoshida, Yuki Ota, Fumihiro Sato, Shayne Morris, Hisato Sugata, Tetsu Goto, Takufumi Yanagisawa, Toshiki Yoshimine

**Affiliations:** ^1^Department of Neurosurgery, Osaka University Medical School, Osaka, Japan; ^2^Department of Mechanical Engineering, Gifu University, Gifu, Japan; ^3^Endowed Research Department of Clinical Neuroengineering, Global Center for Medical Engineering and Informatics, Osaka University, Osaka, Japan; ^4^Center for Information and Neural Networks, National Institute of Information and Communications Technology, Osaka, Japan; ^5^Department of Semiconductor Electronics and Integration Science, Hiroshima University, Hiroshima, Japan; ^6^Department of Electrical Engineering, Graduate School of Engineering, Tohoku University, Miyagi, Japan; ^7^Faculty of Welfare and Health Science, Oita University, Oita, Japan

**Keywords:** brain–machine interface, electrocorticogram, implantable device, bio-signal acquisition, human–machine interface

## Abstract

Brain–machine interfaces (BMIs) are promising devices that can be used as neuroprostheses by severely disabled individuals. Brain surface electroencephalograms (electrocorticograms, ECoGs) can provide input signals that can then be decoded to enable communication with others and to control intelligent prostheses and home electronics. However, conventional systems use wired ECoG recordings. Therefore, the development of wireless systems for clinical ECoG BMIs is a major goal in the field. We developed a fully implantable ECoG signal recording device for human ECoG BMI, i.e., a wireless human ECoG-based real-time BMI system (W-HERBS). In this system, three-dimensional (3D) high-density subdural multiple electrodes are fitted to the brain surface and ECoG measurement units record 128-channel (ch) ECoG signals at a sampling rate of 1 kHz. The units transfer data to the data and power management unit implanted subcutaneously in the abdomen through a subcutaneous stretchable spiral cable. The data and power management unit then communicates with a workstation outside the body and wirelessly receives 400 mW of power from an external wireless transmitter. The workstation records and analyzes the received data in the frequency domain and controls external devices based on analyses. We investigated the performance of the proposed system. We were able to use W-HERBS to detect sine waves with a 4.8-μV amplitude and a 60–200-Hz bandwidth from the ECoG BMIs. W-HERBS is the first fully implantable ECoG-based BMI system with more than 100 ch. It is capable of recording 128-ch subdural ECoG signals with sufficient input-referred noise (3 μV_rms_) and with an acceptable time delay (250 ms). The system contributes to the clinical application of high-performance BMIs and to experimental brain research.

## Introduction

Multiple diseases and symptomatic states lead to the loss of muscular control without disruption of the patient’s cognitive ability. These include amyotrophic lateral sclerosis (ALS), brainstem stroke, spinal cord injury, muscular dystrophy, Parkinson’s disease, and cerebral palsy. The brain–machine interface (BMI) is a clinically promising technology that enables the control of machines in the absence of physical contact with input devices and with direct use of brain signals. Thus, BMI technology can improve independence and quality of life in patients with the above-described conditions by enabling the control of external devices for communication with others and manipulation of their surroundings according to their will.

Electrocorticogram (ECoG) recording and intra-cortical recording are the two major methods used in clinical BMI devices. ECoG recording is commonly used in medical treatments for intractable epilepsy, and recent studies have demonstrated the decoding of arm trajectories (Pistohl et al., 2008; Schalk et al., 2008; Nakanishi et al., 2013; Bundy et al., 2016) and finger movements (Scherer et al., 2009; Nakanishi et al., 2014) using human ECoG signals. ECoG signals have also been recorded using subdural grid electrodes placed on the monkey sensorimotor cortex. These signals were used to stably decode 3D arm positions for nearly 1 year (Chao et al., 2010), indicating that ECoG signals offer reliable information and stable measurements.

Intra-cortical recordings from needle electrodes have been commonly used in the field of neurophysiology. In particular, intra-cortical micro-electrode arrays (Hochberg et al., 2006, 2012; Velliste et al., 2008; Fraser et al., 2009; Collinger et al., 2013; Hiremath et al., 2015) have greatly contributed to the understanding of brain mechanisms, as the arrays offer high temporal and spatial resolution. These arrays have also been used in human and monkey experiments with BMIs (Hochberg et al., 2006, 2012; Velliste et al., 2008; Fraser et al., 2009; Collinger et al., 2013; Hiremath et al., 2015) and have been used to produce robot arm control in real-time. However, these arrays have a risk of gradual reduction in electrode sensitivity due to chronic inflammatory tissue reactions. For this reason, it is not practical to use intra-cortical measurements for clinical BMIs in Japan and some other countries. We thus focused on real-time robot arm control achieved using ECoG-based BMIs (Yanagisawa et al., 2009, 2011, 2012) and the development of a fully implantable wireless recording device (Hirata et al., 2011) in this study.

The BMI system used to achieve real-time robot arm control in this study comprises a commercial 128-channel (ch) digital electroencephalogram (EEG) system, an analytical workstation, and a robot arm. The analytical workstation can decode the simple motor tasks of hand and arm movements from human ECoG signals using fast Fourier transformation (FFT) and a support vector machine (SVM) (Kamitani and Tong, 2005). Subsequently, the robot hand (Yokoi et al., 2009) performed the same movements as the decoded movements, and the correspondence between human and robot hand movements was approximately 70–90%. However, this system uses a wired ECoG recording device, and skin penetration is associated with a risk of infection with prolonged periods of implantation. Therefore, in the present study, we developed a fully implantable wireless ECoG recording system.

Previously developed implantable devices for BMIs include an intra-cortical signal recording device (Borton et al., 2013; Yin et al., 2013) and the epidural ECoG recording device WIMAGINE (Charvet et al., 2013; Mestais et al., 2015). Both of these devices were tested using recordings of invasive brain signals in monkeys. Concomitantly, we have been developing a subdural ECoG recording device since 2009. Our first prototype, which was developed in 2011 (Hirata et al., 2011), was designed to evaluate the system integration of the ECoG recordings and the wireless power supply. In the present study, we report improvements in the size of the wireless data transfer and the wireless power supply, and in the speed of the data transfer.

## Whole System

**FIGURE 1:**
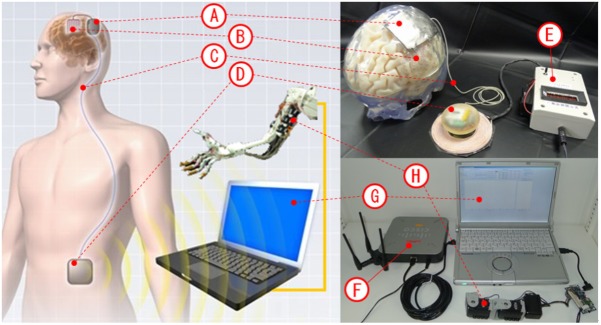
Images of the implantable device. **(A)** Titanium skull casing. **(B)** 3D high-density multiple electrodes. **(C)** Stretchable spiral cable. **(D)** Data and power management unit. **(E)** Wireless power supply (transmitter). **(F)** Wi-Fi access point. **(G)** ECoG monitoring software. **(H)** Example of assistant device.

The design and appearance of the fully implantable wireless 128-ch ECoG recording system (wireless human ECoG-based real-time BMI system, W-HERBS) is shown in **Figure 1**. It is designed to have components implanted in both the head and abdominal locations. The assembly in (A) and (B) is referred to as the head device, and that in (D) is referred to as the abdominal device. The titanium skull casing (A) contains two ECoG measurement units and works as an artificial skull bone replacement for the opened skull bone flap. The 3D high-density multiple electrodes (B) are placed directly on the brain surface and are fitted to the contour of the brain surface. A stretchable spiral cable (C) connects the head and abdominal devices. The data and power management unit in the abdomen (D) communicates with the ECoG measurement units and external devices such as a Wi-Fi access point (F) and a wireless power supply (E). The ECoG monitoring software on the workstation (G) receives ECoG data and transmits control parameters for the ECoG measurement units (E). The wireless power supply transmits power in alternating current (AC) waveform. The details of the device are described in the following sections.

### 3D High-Density Multiple Electrodes

**FIGURE 2:**
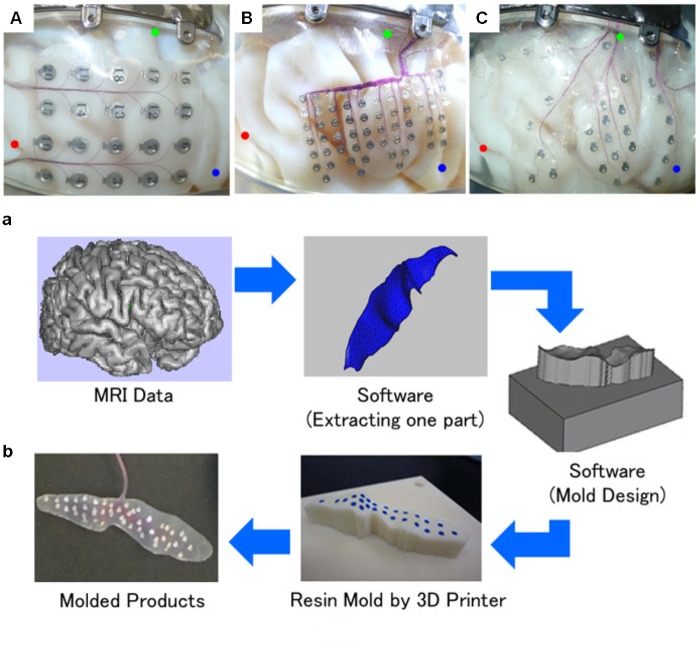
3D high-density multiple electrode sheets. **(A)** The three types (a, b, and c) of multiple electrodes placed on the brain surface model (the three *circles* refer to positions on the brain). **(B)** Manufacturing process.

The 3D high-density multiple electrodes used in this study were designed to improve recording yields and spatial resolution when compared to conventional electrodes used to record ECoG signals. **Figure 2A** illustrates the differences between conventional electrodes (a) and the proposed high-density electrodes (b and c). The contact area of the conventional electrodes is 3.0 mm in diameter and the inter-electrode spacing is 10 mm, while the contact area of the proposed high-density micro-electrodes is 1.0 mm in diameter and the inter-electrode spacing can be up to 2.5 mm. It is known that ECoG signals are produced due to cortical activity in the 2–3-mm area surrounding the electrodes (Slutzky et al., 2010). Accordingly, the inter-electrode spacing of the proposed electrodes provides adequate density to fully utilize the potential of ECoG signals.

The proposed electrodes have almost perfect electrode contact with the folds of the cortex. We developed a novel rapid manufacturing process using 3D printing technologies to press silicon electrode sheets into 3D shapes that closely fit individual cortical surfaces. The detailed manufacturing process is described in our previous paper (Morris et al., 2015) and is presented in **Figure 2B**. This process is comprises the following steps: (1) production of thin-slice brain magnetic resonance imaging (MRI) data (digital imaging and communication in medicine, DICOM format), (2) construction of 3D brain models based on MRI data using the software Mimics (3-matic v5.1, Materialise Inc., N.V.; Leuven, Belgium), (3) extraction of target cortical areas from the model and to design sets of mold models for pressing silicon sheets using the 3D computer-aided designing (CAD) software 3-matic (Mimics v14.12, Materialize Inc., N.V.), (4) fabrication of resin molds using a 3D printer (Polyjet 3D printer, Objet Geometries Ltd., Israel), (5) pressing of silicon sheets using the molds, (6) placement of electrodes at target points on the sheet and to cover them with another silicon sheet, and (7) removal of the electrode sheet from the molds after confirming the fixed adhesion of the covering silicon sheet. This process was successfully used to fit 3D multiple electrodes to target cortical surfaces. There was a wide availability of electrode configurations for effective recording of ECoG signals using individually located electrodes on the CAD software or on the mold. The proposed electrodes (**Figure 2A**; b and c) have different uses. The high-density type (b) is suitable for recording activity from areas in which high spatial resolution is necessary, while the high-density type (c) covers a wide area.

### ECoG Measurement Unit

We developed an analog front-end (AFE) chip (Yoshida et al., 2010, 2011) to acquire target ECoG signals. This chip amplifies 64-ch ECoG signals, performs band-pass filtering, and converts analog voltage signals to 12-bit digital data at a maximum sampling rate of 1 kHz. The specifications of this 5.18 × 5.18-mm chip are listed in **Table 1**.

**Table 1:** Chip specifications.

Parameter	Specification
Number of input channels	64
Low pass filter	0.1/1/10 Hz
High pass filter	240/500/1,000 Hz
Amplifier gain	40/50/60/70/80 dB
Input voltage range	1 μV to 1 mV
Sampling rate	200/500/1,000 Hz
Power consumption	5 mW
Noise	10 μV

**FIGURE 3:**
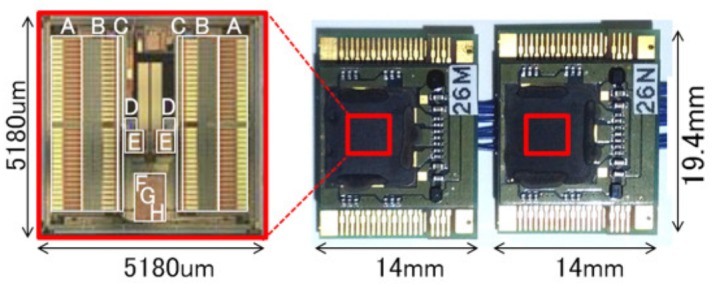
Appearance of ECoG measurement units: two units for 128 ch. The *red square* indicates one chip for 64 ch.

As shown in **Figure 3**, two AFE chips are placed on two circuit boards (14.0 mm × 19.4 mm × 2.5 mm) and are connected via flexible printed circuits that comprise a 128-ch unit (ECoG measurement unit). Each board has 73 electrode pads for ECoG measurements and eight pads for chip control. The 73 electrode pads include 64 pads for ECoG signals, eight pads for reference signals (each pad is associated with eight ECoG signal pads), and one pad for ground.

In the present prototype, 128-ch electrodes were connected to the pads separately, a common reference electrode was connected to all 16 reference pads, and a ground electrode was installed. The eight control signal pads were connected to the data and power management unit using stretchable spiral cables. The eight control signals were carried using two lines for power (VCC and GND), four lines for control input (CLK, RST, SDI, and WTE), and two lines for data output (TRO and SDO). Overall, 10 lines were required to relay the 128-ch ECoG signals because the two ECoG measurement units shared the two power lines with the four control input lines, but not with the two data output lines.

### Titanium Skull Casing

The titanium skull casing was designed to hermetically shield ECoG measurement units against environmental noise, to seal tissue fluids, and to protect from external impact. It was also designed to act as an artificial skull bone to replace the skull bone flaps removed during craniotomy.

**FIGURE 4:**
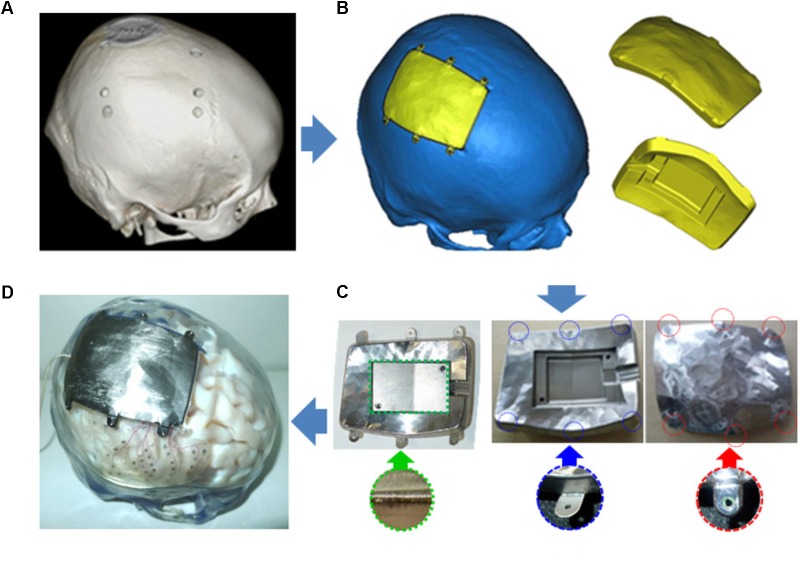
Manufacturing process for the titanium skull casing. **(A)** 3D model construction based on CT scan. **(B)** Skull casing designed by editing the 3D model. **(C)** Machining and laser-welding operation for a skull casing. **(D)** Example of implantation.

The manufacturing process (**Figure 4**) comprised the following steps: (1) obtaining a thin-slice skull bone computed tomography (CT) scan, (2) construction of a 3D skull model using CT data (DICOM format) and 3D CAD software (Mimics), (3) extraction of a craniotomy area from the model, designing the casing model, and laying out the space used to house the ECoG measurement unit using 3D CAD software (3-matic), (4) planning the machining processes based on the 3D CAD data using CAD and computer aided manufacturing software GibbsCAM^®^ (Gibbs and Associates, CA, United States), (5) cutting out the casing model from a titanium block using a 5-axis machining center, (6) laser-welding titanium screw-hole parts to fix the casing to the craniotomy site of the skull, (7) laser-welding the titanium plate for hermetic sealing after placement of the ECoG measurement units in the layout space, and (8) filling the space with epoxy resin.

### Flexible and Stretchable Spiral Cable

The flexible and stretchable spiral cable connects the ECoG measurement unit in the skull casing with the data and power management unit in the abdominal casing, and runs subcutaneously from the skull casing to the abdominal casing. The spiral cable is designed based on a deep brain stimulator (DBS) cable: it comprises 10 super-fine coaxial cables (AWG42) that are spirally fixed within a urethane resin tube (ϕ2 mm × 900 mm).

### Abdominal Device

**FIGURE 5:**
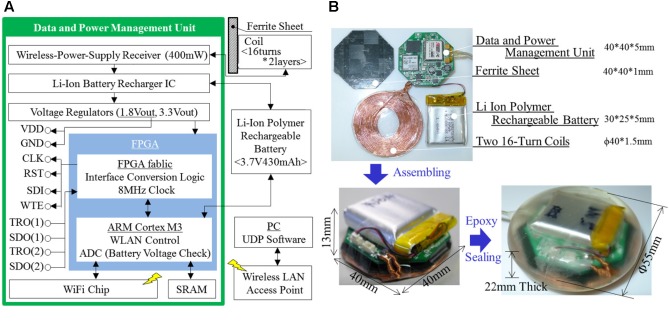
Abdominal device, including a data and power management unit, a lithium ion polymer rechargeable battery, a ferrite sheet, coils, and epoxy seal. **(A)** Schematic architecture. **(B)** Appearance.

The abdominal device comprises a data and power management unit, a lithium ion polymer rechargeable battery, a ferrite sheet, two 16-turn coils, and an epoxy resin seal. The data and power management unit is the main component of this device and contains the field-programmable gate array chip Microsemi A2F200M3F-CS288, the Wi-Fi chip Redpine Signals RS9110-N-11-24, and an original wireless power supply receiver circuit (**Figure 5A**). The Wi-Fi chip was added due to a prospective medical wireless communication protocols. The unit transfers control parameters including gain, filtering information, and recording frequencies from the software to the ECoG measurement units, and data (12 bits/ch × 128 ch × 1 kHz) from the ECoG measurement units to the software. The unit is powered (200 mW) by a battery without a wireless power supply. The entire unit and battery use 400 mW during wireless charging from the power supply. The casing is made of epoxy resin in order to isolate the electrical parts from biological organs. It provides bio-compatibility for animal tests, but not human implantation. We plan to use commercial hermitically sealed technology for the next prototype. The abdominal device is shown in **Figure 5B**.

### Wireless Power Supply

**FIGURE 6:**
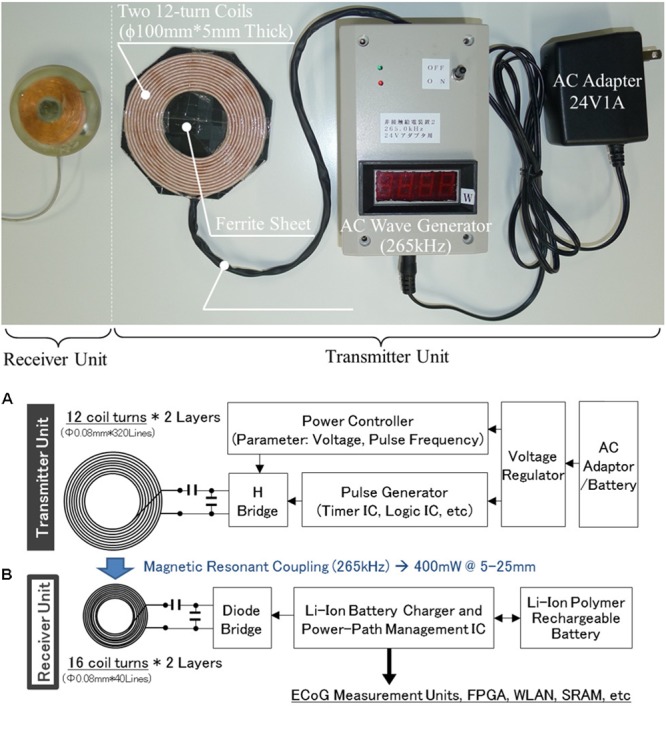
Specifications of the wireless power supply. **(A)** Appearance. **(B)** Schematic architecture.

The wireless power supply comprises a set of receiver units in the abdominal device and a transmitter unit outside the body (**Figure 6**). Each unit comprises a circuit, a ferrite sheet, and coils. The transmitter circuit generates magnetic radiation in AC waveform, and the receiver circuit converts the received magnetic radiation to direct current. Ferrite sheets protect the circuits from the transmitted magnetic radiation. Copper strand wires are used as coil material, and the magnetic resonance coupling method is used to improve the efficiency of power transfer between coils. The magnetic frequency is set to 265 kHz to assure safety. Because the coil area of the transmitter unit (100 mm × 100 mm) is greater than that of the receiver unit (40 mm × 40 mm), it offers a wide power transmission area to accommodate the invisibility and rough estimation of the positioning of the implanted receiver unit. Finally, 400 mW from the transmitter unit (24 V) to the receiver unit (5 V) over a distance of 5–25 mm is sufficient to power the whole device (200 mW) and to recharge the battery (200 mW).

### Graphical User Interface and Character User Interface Data Monitoring Software

We used data monitoring software packages including a graphical user interface (GUI)-style program to monitor real-time data during certain periods and a functioning FFT analysis and motor control program for the BMI. Character user interface (CUI)-style programs for long-term recording are also suitable for neuroscience studies, such as night recordings of ECoG.

The GUI software communicating with the implantable device enables adjustment of transmission control parameters, such as gain, band-pass filter settings, and amplification. This software receives 128-ch signals and displays the signals and FFT data from three selected channels.

The CUI software was designed to record data without data losses and has only three functions, as follows: waiting for data from implantable devices at all times, saving received data into binary files as quickly as possible, and transfer of received data to other computers using serial communication as quickly as possible. The ensuing direct transfer allows other functions such as motor control to be performed by other workstations and micro-chip computers. During the performance investigation, we recorded data for 24 h using the CUI software. The recorded files contained all time stamps and data amounts, and reflected voltage continuity. There were no data losses.

## Performance Investigation

Performance of the W-HERBS device in the following five domains was evaluated using engineering methods: feasibility for implantation in 3D human models (See Experiment 1: Feasibility for Human Implantation), operating temperature and time (See Experiment 2: W-HERBS Operating Characteristics), noise levels and recording performance compared to the conventional measurement device (See Experiment 3: Noise Levels and Recording Performance), frequency analysis (Experiment 4: Detection of Frequency Components for ECoG BMI), and real-time recording (See Experiment 5: Real-Time Performance).

### Experiment 1: Feasibility for Human Implantation

The present W-HERBS prototype is intended for future human implantation. Therefore, we evaluated the feasibility of fitting the original manufactured components to the human body in terms of material, size, and shape. The components in direct contact with tissue include the 3D high-density multiple electrodes and the titanium skull casing, the stretchable cable, and the casing of the abdominal device. All components were produced using materials with medically demonstrated biocompatibility.

The electrodes and skull casings were especially manufactured according to individual CT and MRI data. For this reason, we also designed 3D brain and skull models to simulate implantation using the same individual CT and MRI data. We then placed the proposed electrodes and skull casing into the models. Eventually, the above components were fitted into the target area with accuracy acceptable to neurosurgeons. Specifically, the electrodes coincided with the target cortex and were easily fixed at a certain position. For installation in subjects with thin skull bones, the casing smoothly protruded outward by several millimeters from the original skull bone to obtain the necessary layout space for the ECoG measurement units.

The abdominal device was designed based on the widely used baclofen pump, which is an abdominal implant with a tank for intra-thecal baclofen infusions. The implant is 75 mm in width and 20 mm in thickness, and weighs 165 g. The abdominal device used here was 55 mm in diameter and 22 mm in thickness. This indicates that our device can be used as an abdominal implant. The stretchable cable was designed based on the DBS cable, as described in Section ***“***Flexible and Stretchable Spiral Cable.”

### Experiment 2: W-HERBS Operating Characteristics

Initially, we confirmed that the maximum operating temperatures of the devices did not cause thermal damage to tissues. It is known that a temperature of 38°C is sufficient to start a low-temperature burn. Of the W-HERBS components, the abdominal device consumes most of the power and generates the most heat. The appearance of the whole abdominal device during operation in still air was photographed using a thermography device (FLIR I7, FLIR Systems Inc., United States). The surficial temperatures of the device did not exceed 38°C, indicating that W-HERBS do not damage tissue.

The maximum battery operation time was calculated based on power consumption by the system and the electrical capacity of the battery. W-HERBS consumes approximately 200 mWh, and the battery has a 1.6-Wh capacity. This results in a maximum operating time of approximately 8 h. In subsequent performance tests, we recorded battery voltage transitions during and after wireless charging. In these experiments, the slowest charging setup was used to facilitate careful observation, and W-HERBS was wirelessly powered for 8.5 h. The W-HERBS lasted for 6 h, thus demonstrating appropriate performance of the wireless power supply and the battery.

### Experiment 3: Noise Levels and Recording Performance

**FIGURE 7:**
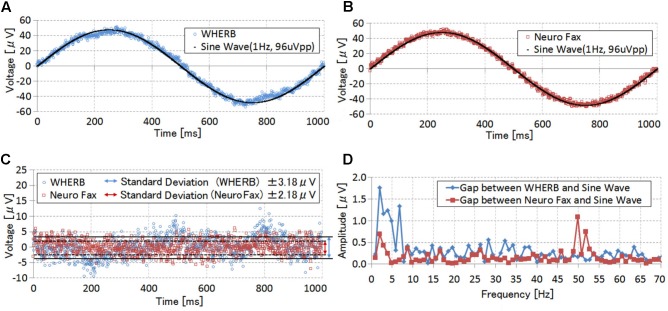
Performance comparisons of sine-wave measurements. **(A)** A signal measured using W-HERBS. **(B)** A signal measured using NeuroFax. **(C)** Gap standard deviations between measured signals and theoretical sine waves. **(C)** Frequency analyses of the gap standard deviations.

We compared signal quality between W-HERBS and the commercial EEG system (NeuroFax, Nihon Koden; Tokyo, Japan) using general engineering methods to confirm that W-HERBS can sufficiently obtain ECoG measurements. A sine wave (frequency, 1 Hz; amplitude, 48 μV) was input to one channel for both devices through electrode wires 100-mm in length by a function generator in an office environment without an electrical shield. We then analyzed the measured signals (**Figure 7**). The proposed sine wave was regarded as the minimum frequency bandwidth for brain wave measurements (i.e., the delta wave has a bandwidth of 1–4 Hz) and enabled us to evaluate input referred noise at bandwidths greater than 1 Hz while measuring a signal.

**Figures 7A,B** show the measured and theoretical sine waves (one cycle of the measured sine wave was cut out and translocated to the same position as the theoretical sine wave). Both figures indicate that the measured signals almost coincided with the theoretical sine waves, with gap standard deviations (**Figure 7C**) of ±3.18 μV and ±2.18 μV for W-HERBS and NeuroFax, respectively. Subsequent FFT analyses of the gaps (**Figure 7D**) revealed that W-HERBS had larger gaps in all bandwidths than NeuroFax did, indicating inferior noise levels. However, the noise level was less than ±1 μV, indicating that W-HERBS can be used as an alternative to NeuroFax in ECoG BMI experiments. W-HERBS also had remarkable gaps for the 3-Hz bandwidth. These gaps reflected periodic noises from the data and power management units during the transfer of mass data at intervals of 300 ms. However, this is not a problem because the 3-Hz bandwidth is used to analyze the delta waves, which have much higher amplitudes than 2 μV. In addition, these waves are outside the target range for ECoG BMI. We observed noise in the 50-Hz bandwidth with NeuroFax, which originated from the commercial household power supply.

### Experiment 4: Detection of Frequency Components for ECoG BMI

**FIGURE 8:**
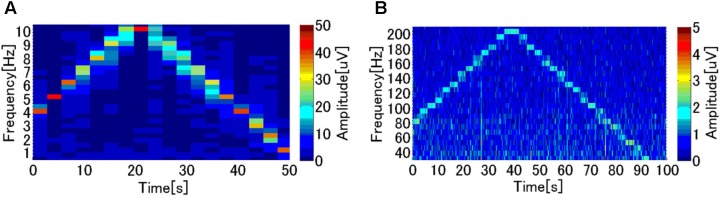
Performance investigation for the ECoG BMI. **(A)** Frequencies of sine wave (48 μV) changes between 1 and 10 Hz with 1-Hz intervals of 3 s. **(B)** Frequencies of a sine wave (4.8 μV) changes between 40 and 200 Hz with 10-Hz intervals of 3 s.

We investigated the detection of frequency components, which determines the classification performance of the SVM. Previous research (Yanagisawa et al., 2009, 2011, 2012; Hirata et al., 2011) on wired ECoG BMI devices indicates that ECoG signals at 5 V with bandwidths of 60–200 Hz and those at 50 μV with bandwidths of 1–10 Hz distinctly represent human motion intentions. Thus, in the present performance investigation of W-HERBS, we used sine wave signals 48 μV in amplitude and lasting for 3 s with repeated fluctuations between 1 and 10 Hz at 1-Hz intervals. We also used sine waves 4.8 μV in amplitude lasting for 3 s with fluctuations between 40 and 200 Hz. Subsequently, these sine waves were recorded at 700-ms intervals, and were analyzed in spectrograms using the open source Fastest Fourier Transform in the World library^1^, which is commonly used for such analyses. Under both conditions (**Figures 8A,B**), a single peak at the target frequency was observed in each step. Because the FFT window displayed two frequencies at the frequency switching time, we sometimes observed wider distributions. However, the peaks clearly formed steps, indicating clear measurements at the 48-μV amplitude in the 1–10-Hz bandwidths and at the 4.8-μV amplitude in the 60–200-Hz bandwidths. Therefore, the performance of W-HERBS was sufficient for ECoG BMI.

### Experiment 5: Real-Time Performance

We assessed the real-time performance of W-HERBS by measuring the time from signal input to the end of the frequency analyses. W-HERBS transmits data every 7 ms, and the workstation sequentially receives data and processes frequency analyses after accumulation of a certain amount of stored data. Specifically, the analysis requires approximately 3 s of stored data to detect 1-Hz signals and approximately 70 ms of stored data to detect 40-Hz signals. Therefore, we set the fastest stored data cycles to 70 ms to measure the time between signal input and the end of the frequency analyses. Average, maximum, and minimum times were calculated based on 20 data points from each time-cycle setup. Eventually, there was little difference in time between the conditions because frequency analyses constantly required 2–3 ms, but access to the User Datagram Protocol buffer on the Windows operating system requires between 160 and 350 ms. In short, the average time delay was 250 ms. For ECoG BMI measurements, a 250-ms time delay is considered relatively fast response and is regarded as real-time, especially as human motion intention appears in ECoG signals appropriately 200 ms before the start of actual body motion.

## Discussion

### Developmental History of W-HERBS

The first W-HERBS prototype was developed in 2011 (Hirata et al., 2011) as an original ECoG measurement unit with the goal of establishing an integrated system of ECoG measurement units with wireless data transfer and power supply. We performed wireless data transfer using Bluetooth, which was the most practical protocol for small devices at that time. We demonstrated transfer of 16-ch ECoG data at a sampling rate of 500 Hz. However, the wireless chip used had the relatively high energy requirement of 4 Wh for wireless power supply and the abdominal device was twice as large as the conventional medical device. We designed an integrated system and demonstrated target performance.

We achieved full data transfer using the second prototype and were able to downsize the wireless power supply using a low-power Wi-Fi chip able to transfer 128-ch ECoG data with a sufficient sampling rate of 1 kHz. The low-power Wi-Fi chip also contributed to a reduction in total power consumption to 400 mW. In addition, the diameter of the abdominal device was reduced from 80 to 40 mm. Finally, we confirmed that the ECoG 128-ch signals were recorded at a sampling rate of 1 kHz.

### Conventional Implantable Devices for BMI

Three previous studies of fully implantable devices for BMIs intended for future clinical application have been published to date. They include the present report regarding the W-HERBS device, one regarding the implantable wireless neural interface developed at Brown University (Borton et al., 2013), and one regarding the WIMAGINE system developed at the CEA-LETI-CLINATEC (Mestais et al., 2015; **Table 2**).

**Table 2:** Comparison of implantable devices for BMI.

Device Name	Brown University’s Device	WIMAGINE	W-HERBS
Target signal	Spike	ECoG	ECoG
Electrode location	Intra-cortical	Epidural	Subdural
Number of channels	100 ch	64 ch	128 ch
Wireless communication	24 Mbps (3.2–3.8 GHz)	450 kbps (402–405 MHz)	1.6 Mbps (2.4 GHz)
Bandwidth	0.1–7,800 Hz	0.5–300 Hz	0.1–500 Hz
Sampling rate	20 kHz	390/585/976/2,900 Hz	250/500/1,000 Hz
Gain	200	1/5/280/990/1,370	100/315/1,000 /3,162/10,000
ADC resolution	12 bits	12 bits	12 bits
ADC power supply	3 V	3.3 V	1.8 V
Coil distance for wireless power supply	20 mm	20 mm	5–25 mm
Input referred noise	8.6 μV_rms_	0.7 μV_rms_	3 μV_rms_
Size	50 mm × 40 mm × 10 mm	50 mm × 50 mm × 10 mm	21 mm × 25 mm × 7 mm Φ55 mm × 22 mm

We obtained full measurements of 128-ch subdural ECoG signals at 1 kHz for 6 h using W-HERBS. The wireless power supply was sufficient for charging at 5–25 mm. The device developed at Brown University was used to wirelessly measure intra-cortical signals from a 100-ch intra-cortical micro-needle electrode array for 6 h, and was capable of wirelessly supplying power at a distance of 5 mm. In contrast, WIMAGINE was designed to measure epidural ECoG signals. This device can be used to measure signals from up to 64 ch and was shown to wirelessly measure 32-ch signals at a sampling rate of 1 kHz for 6 h with a wireless power supply at a distance of 20 mm.

### Comparison: Design Policy

The main differences between current BMI devices stem from the target signal. Although the device from Brown University is designed to measure intra-cortical signals and has the fastest sampling rates and lowest amplifications, both W-HERBS and WIMAGINE devices are designed to measure ECoG signals and therefore have similar sampling rates and gains. However, the electrodes of W-HERBS are subdurally placed and measure subdural ECoG signals, while those of the WIMAGINE system have epidural placement and measure epidural ECoG signals.

### Comparison: Measurement Stability and Safety

The data obtained here using W-HERBS indicate that its feasibility for human implantation and signal acquisition performance may be superior to those of the other ECoG BMI systems. Moreover, W-HERBS uses a subdural electrode placement similar to that utilized in common medical devices used to identify epileptic foci in patients with intractable epilepsy. Because the subdural space is protected by the blood–brain barrier, the electrodes are hardly exposed to chronic inflammation and produce long-term stable measurements. In contrast, the device developed at Brown University uses an intra-cortical micro-needle electrode array (100 ch), which is inserted into the cerebral cortex and offers the highest signal quality. Nonetheless, the invasiveness of the device tends to cause chronic tissue inflammation and leads to gradual deterioration in signal quality (Polikov et al., 2005; Winslow and Tresco, 2010; Hochberg et al., 2012). Therefore, intra-cortical measurements cannot be used for clinical BMI devices in Japan and some other countries at present. The electrodes of the WIMAGINE system are placed epidurally. However, the epidural space is outside of the blood–brain barrier. This results in exposure of the electrodes to chronic inflammation, which lead to gradual deterioration in signal quality over several months (Schendel et al., 2013). Thus, epidural ECoG may potentially produce lower quality signals than subdural ECoG.

### Originality of W-HERBS

W-HERBS is the first fully implantable device to record 128-ch subdural ECoG signals. The 3D high-density multiple electrodes used here are precisely fitted to the cerebral cortex and stably record subdural ECoGs from target cerebral areas (Morris et al., 2015). Moreover, these electrode arrays can maintain contact with the entire area of the hand motor cortex (approximately 20 mm × 20 mm) with 2.5-mm inter-electrode spacing and provide sufficient information for hand movement assistance in real-time. Finally, W-HERBS is regarded as the first fully implantable ECoG BMI system able to detect high gamma bandwidth signals at 4.8-μV amplitude and control motor movements based on data analyses.

### Future Development of W-HERBS

The second prototype of this device comprises a head device and an abdominal device because data and power management units have relatively high-power requirements. As a result, minimizing the size of the battery for incorporation into the head device precluded its operation for 6 h. Further reduction of power consumption using a lower-power wireless data transfer module will be required in the future (i.e., 60 mW for 24-h continuous operation with a 1.6-Wh battery).

Further development requires practical bio-compatibility achieved using hermetically sealed casings to isolate the electrical parts of our human-implantable device from biological organs. The technology is already commercially available and will be used for both the skull and abdominal casings in future studies. Accordingly, our third prototype will have fixed basic electrical components and arrangements, and a preset shape for the titanium casing, which would in turn allow the use of such hermetic seals.

## Conclusion

We developed a novel fully implantable wireless 128-ch recording human ECoG BMI device. This W-HERBS device comprises 3D high-density multiple electrodes that can be fitted to individual cortical shapes, ECoG measurement units that amplify and digitize ECoG signals at 1 kHz and transmit data, a titanium casing that fits the removed skull area, a stretchable spiral cable subcutaneously connecting the head and abdominal devices, a data and power management unit that communicates with a workstation using Wi-Fi and supplies 400-mW power over 5–25 mm, and software for recording and analysis of the transferred data. We demonstrated that W-HERBS sufficiently performed measurements for ECoG BMI, producing data that can be analyzed based on sine waves 4.8 μV in amplitude with a 60–200-Hz bandwidth.

## Ethics Statement

The study was approved by the Hamamatsu Pharma Research Ethics Committee (Approval Number: PR0098-1; Approval Date: November 19, 2012; Contents: Consent procedure used for animal owners).

## Author Contributions

KM is the main research conductor: making device specification, assembling, and performance investigation, and paper writing. MH, SM, TG, TYa, and ToY are the medical doctors and HS is a physical therapist and those designed the system from medical aspects: they designed the skull case design and conducted animal experiments. TS, HA, and TYo are in charge of the semiconductor fabrication and wireless data communication circuits. YO and FS built wireless power supplies and investigated the performance.

## Conflict of Interest Statement

The authors declare that the research was conducted in the absence of any commercial or financial relationships that could be construed as a potential conflict of interest.

## Footnotes

1 http://www.fftw.org/
